# Overview of cancer incidence and mortality among people living with HIV/AIDS in British Columbia, Canada: Implications for HAART use and NADM development

**DOI:** 10.1186/s12885-017-3229-1

**Published:** 2017-04-14

**Authors:** Connie G. Chiu, Danielle Smith, Kate A. Salters, Wendy Zhang, Steve Kanters, David Milan, Julio S.G. Montaner, Andy Coldman, Robert S. Hogg, Sam M. Wiseman

**Affiliations:** 1 Department of Surgery, St. Paul’s Hospital, & University of British Columbia, C303 – 1081 Burrard Street, Vancouver, BC V6Z 1Y6 Canada; 2 Faculty оf Health Sciences, Simon Fraser University, Burnaby, BC Canada; 3 British Columbia Centre For Excellence In HIV/AIDS, Providence Health Care, St. Paul’s Hospital, Vancouver, BC Canada; 4 Faculty of Medicine, University of British Columbia, Vancouver, Canada; 5 Population and Preventive Oncology, British Columbia Cancer Agency, Vancouver, BC Canada

**Keywords:** Cancer, HIV, Aids, Epidemiology, HAART, Malignancy

## Abstract

**Background:**

The objective of this study is to evaluate the incidence of non-AIDS defining malignancies (NADMs) among people living with HIV/AIDS (PLWHA) in British Columbia, focusing on clinical correlates, highly active antiretroviral therapy (HAART) use, and survival, in order to elucidate mechanisms for NADM development.

**Methods:**

A retrospective population based analysis was carried out for individuals with HIV/AIDS that began their treatment between 1996 and 2008.

**Results:**

There were 145 (2.95%) NADMs and 123 (2.50%) AIDS defining malignancies (ADMs) identified in 4918 PLWHA in the study population. NADMs were represented by a range of cancer types including, most commonly, lung cancer, followed by anal, breast, head/neck, prostate, liver, rectal, and renal cancers. PLWHA had a SIR of 2.05 (CI:1.73, 2.41) for the development of NADMs compared to individuals without an HIV/AIDS diagnosis in the general population. Independent factors significantly associated with a NADM were: male gender, older age, lower CD4 cell counts, previous NADM, absence of HAART (non-HAART versus HAART) and treatment during the early-HAART era (before 2000 versus after 2000).

**Conclusions:**

NADMs represent an important source of morbidity for PLWHA. Use of HAART with its associated improvement in immune-restoration, and tailored targeted cancer screening interventions, may be beneficial and improve outcomes in this unique patient population.

## Background

The advent of combination triple antiretroviral therapy (ART), later referred to as highly active ART ﻿(HAART), was found to result in superior patient outcomes and sustained HIV suppression [1, 2]. Reports of remarkable improvements in patient survival and morbidity with the expanded use of the HAART regimen soon followed, ushering in a new era in which HIV/AIDS became viewed as a chronic manageable disease [3–6]. The longevity experienced by people living with HIV/AIDS (PLWHA) in the modern HAART era has resulted in increasing vulnerability to age-related diseases and new health challenges [7, 8]. The decline in the incidence of AIDS defining malignancies (ADMs) (Kaposi’s sarcoma, non-Hodgkin lymphoma, and invasive cervical cancer) has been accompanied by a dramatic increase in the incidence of non-AIDS defining malignancies (NADMs) [9–11]. However, the incidence of NADMs amongst PLWHA does not appear to be attributable to age alone, as PLWHA have an increased risk of malignancy compared to age-matched cohorts in the general population. While behavioural differences between HIV-positive and HIV-negative populations must be noted, studies controlling for smoking status found PLWHA to remain at increased risk for lung cancer development when compared to the general population [12, 13]. Furthermore, as well as an increased risk of viral co-infection (such as Hepatitis viruses, Human Papillomaviruses, and the Epstein-Barr virus) [14–18] and incidence of cancers attributable to these tumor-associated oncogenic viruses, PLWHA also have a higher incidence of cancer types that have no known viral etiology, notably lung cancer [19]. Finally, cancer-related outcomes may vary by HIV serostatus as NADM related mortality is observably higher compared to the general population [20]. Thus, fundamental characteristics reflective of the immune and health status of PLWHA represent important factors that may mediate the differential cancer risk observed in the HIV/AIDS population.

This is amongst the first Canadian studies reporting on NADMs in PLWHA. It also represents one of only a few contemporary reports evaluating cancer risk during the late-HAART era. Our study is uniquely poised because antiretroviral medications are centrally administered and are free of charge in British Columbia, Canada, resulting in a comprehensive population based database of all individuals receiving antiretroviral therapy in the province. Thus, our study captured detailed drug information and reduces confounding bias related to access to care. The objective of this study was to determine the incidence, clinical correlates, and survival outcomes of NADMs amongst PLWHA in British Columbia, Canada, during the HAART era.

## Methods

### Study design and data sources

This retrospective study utilized a linkage of health administrative data from two comprehensive population health databases in Bristish Columbia, Canada. The British Columbia Cancer Registry (BCCR) is a province-wide mandatory reporting database that records all new cancer diagnoses in British Columbia. The BCCR catalogues individual demographic, cancer specific and mortality data for all cancer patients in the province. The HIV/AIDS Drug Treatment Program (DTP) Registry was established by the British Columbia Centre for Excellence in HIV/AIDS (BC-CfE). All antiretroviral medications are distributed at no cost to all individuals with an HIV/AIDS diagnosis living in British Columbia through a centralized single-payer system and are recorded in the program registry. The registry was created to monitor trends and regional differences in access to antiretroviral therapy and to evaluate the success of treatment on reducing HIV-related morbidity and mortality in British Columbia. The DTP Registry contains individual administrative records of patient antiretroviral drug regimens as well as demographic, clinical, and laboratory data. The BCCR and the DTP Registry are expected to provide a comprehensive listing of all individuals with a cancer diagnosis and all individuals with an HIV/AIDS diagnosis undergoing antiretroviral therapy, respectively, in British Columbia. This study was approved by our institutional research ethics board.

### Data linkage

A probabilistic-match procedure based on Personal Health Numbers (PHNs), names, and dates of birth contained in the BCCR and DTP registries was carried out to generate a province-wide listing of all individuals with an HIV/AIDS diagnosis that had or had not been diagnosed with cancer. This cohort was then enriched by the DTP database with antiretroviral related treatment information. All individual names and identifying information were removed from the resulting datasets before being provided to the study data analysts at the BC-CfE. Analyses were then carried out on the aggregate individual level anonymized dataset.

### Study criteria

Individuals in the DTP registry were included in the study if their antiretroviral therapy was initiated between August 1^st^, 1996 and March 31^st^, 2008. The study start date was chosen to reflect the beginning of widespread use of HAART in British Columbia. Furthermore, the study also separates individuals into the early-HAART (1996–2000) and late-HAART (2000 and later) eras, distinguished by the introduction of a more effective HAART regimen in 2000. Patients were excluded if they were aged 18 years or younger at initiation of antiretroviral therapy. All cases of cutaneous squamous cell carcinoma and basal cell carcinoma were excluded due to suspected non-uniform reporting for these cancers as cases may be topically treated without pathologic confirmation or registry reporting. Follow-up time was until December 31^st^, 2008. Both NADMs and ADMs diagnosed after 1996, regardless of HAART status, were evaluated, as were predictors of NADM while receiving treatment. While a few individuals in the study cohort were diagnosed with multiple cancer types, only the first NADM and ADM that was diagnosed prior to, and after, the initiation of HAART therapy was entered into the study database.

### Statistical analyses

To calculate the standardized incidence ratios (SIR), indirect method of adjustment for age and sex was used. We applied BC population (as our standard population via the BCCR) cancer rates to our study sample to determine expected counts, then we used ‘observed count/expected count’ to determine the standardized incidence rate (SIR). Yearly population incidence rates were available from 1996 to 2003 and for 2007. Expected cases for 2004 and 2005 were based upon incidence rates for 2003, and expected cases for 2006 through 2008 were obtained from the 2007 incidence rates.

The study cohort was used to evaluate the determinants of NADM incidence. The variables evaluated in these analyses included: gender, age at enrollment in DTP, nadir CD4 level, baseline viral load, history of intravenous drug use, presence of ADM, presence of Hepatitis C infection, use of HAART (non-HAART versus HAART), and HAART era at start of antiretroviral therapy (early-HAART versus late-HAART, i.e. prior to 2000 versus 2000 and later). HAART was defined as use of combination triple-antiretroviral therapy, and non-HAART was defined as use of mono- or dual-antiretroviral therapy. Contingency tables were constructed utilizing Fisher’s exact test for evaluation of association between variables and cancer incidence. Logistic regression was then utilized to determine the variables that were independent of the development of NADMs. As these were explanatory models, the model selection was performed by minimizing the Akaike information criterion (AIC) while limiting the type III *p*-values to less than 0.20.

Kaplan-Meier product limit estimates of cumulative overall survival were obtained for NADM cases in the study cohort. Patients contributed to time at risk (in person-years) from start of therapy to either date of NADM diagnosis or date of censoring. Patients were censored because they had: no NADM by the end of the study period, moved out of province, or were lost to follow-up. All individuals in the DTP cohort, including those individuals diagnosed with an ADM, contributed to the person-time calculation.

## Results

The demographic and clinical characteristics of HIV/AIDS patients in the study population are summarized in Table 1. The cohort consisted of 4918 men and women. The median follow-up time was 64 months (Interquartile range (IQR): 32 to 112 months). Study participants were more likely to be men, younger than 50 years, and have a nadir CD4 level lower than 200 cells/mm3. The majority of the study HIV/AIDS population did not have an ADM at the start of the study period upon initiation of antiretroviral therapy. All patients in the study cohort received antiretroviral drug therapy, with the majority of these individuals receiving HAART (combination triple-therapy), and only a few patients receiving non-HAART therapy (mono- or dual-antiretroviral therapy).

**Table 1:** Clinical characteristics of PLWHA in the study population (*n* = 4918)

Clinical characteristic	Number (Percent) of Individuals
Gender	
Male	4016 (81.7%)
Female	902 (18.3%)
Age (years) at Start of Anti-retroviral Therapy	
Under 30	697 (14.1%)
30–39	1932 (39.3%)
40–49	1519 (30.9%)
50 or more	770 (15.7%)
Nadir CD4 Level	
Less than 50	1402 (28.5%)
50–99	686 (13.9%)
100–199	1430 (29.1%)
200 or more	1400 (28.5%)
Baseline Viral Load	
(Log base 10)^a^	4.95 (4.40–5.00)
Intravenous Drug Use	
Yes	1766 (35.9%)
No	3152 (64.1%)
ADM Status	
Yes	251 (5.1%)
No	4667 (94.9%)
Hepatitis C Status	
Positive	2014 (41.0%)
Negative	2110 (42.9%)
Unknown	794 (16.1%)
Use of HAART^b^	
Yes	4275 (86.9%)
No	643 (13.1%)
Start of Antiretroviral Therapy^c^	
Early-HAART era (before 2000)	2093 (42.6%)
Late-HAART era (2000 and after)	2825 (57.4%)

^a^Median and Interquartile Range (IQR)

^b^All individuals were treated with antiretroviral therapy (monotherapy, dual therapy or triple therapy); selected individuals were treated with HAART (triple therapy) according to guidelines at time of treatment

^c^The year cut-point was utilized to represent use of more efficient HAART starting in the year 2000

### Incidence of non-AIDS defining malignancies in study HIV/AIDS population

During the study period, 145 cases (2.95%) of NADMs and 123 cases (2.50%) of ADMs were diagnosed in 4918 PLWHA. These cases represent individuals that were all receiving antiretroviral therapy at the time of their cancer diagnosis. The NADMs there were diagnosed represented a range of cancer types and included cancers of the head and neck, breast, lung, gastrointestinal tract, genitourinary system and skin (Table 2). Of the NADMs diagnosed during the study period, the most common cancer sites were lung (20.7%) and anus (20.0%), followed by head and neck, liver, rectum, prostate, kidney and lymphatic system (>5% each). However, lung and anal cancers combined represented less than half of all NADMs, and PLWHA were at increased risk for a variety of other NADM types.

**Table 2:** NADMs identified in PLWHA receiving antiretroviral therapy by cancer type (*n* = 145)

Cancer type	Number of cases (% of NADMs)	Incidence rate (per 100,000 person-years)
Breast	5 (3.4)	100.03
Lung	30 (20.7)	104.97
Gastrointestinal		
Liver	8 (5.5)	27.99
Gastric	2 (1.4)	6.99
Colon	3 (2.1)	10.50
Rectum	8 (5.5)	27.99
Anal	29 (20.0)	101.47
Genital		
Vulva	3 (2.1)	60.02
Testicle, scrotum	3 (2.1)	12.72
Prostate	9 (6.2)	38.17
Urinary		
Bladder	2 (1.4)	6.99
Kidney	8 (5.5)	27.99
Skin		
Melanoma	2 (1.4)	6.99
Other		
Unknown primary	5 (3.4)	17.50
Soft tissue	2 (1.4)	6.99
Brain, spinal cord	2 (1.4)	6.99
Hematologic	5 (3.4)	17.50
Lymphatic system	10 (6.9)	34.99

Total: 145 cases, 28,579.05 person-years

Female: 4998.45 person-years

Male: 23,580.60 person-years

The incidence of NADMs in our PLWHA study cohort was compared to their expected incidence in the general population (Table 3). Individuals with an HIV/AIDS diagnosis had a SIR of 2.05 (95% confidence interval [CI]: 1.73 to 2.41) for the development of NADMs compared to individuals not diagnosed with HIV/AIDS in the general population. A particularly large effect size was observed in younger men with HIV/AIDS (age 20 to 39), who had a SIR of 5.42 (CI: 3.31 to 8.38) for NADMs. A sub-group SIR analysis was carried out for the most frequently diagnosed NADM cancer types (Table 3). Cancers of the lung and anal canal had a significantly higher incidence in PLWHA compared to the general population.

**Table 3:** Standardized incidence ratio of NADMs in PLWHA on antiretroviral therapy

Age/Sex group	Number of NADM		Standardized Incidence ratios (95% CI)
	Actual	Expected^a^	
All NADMs			
20–39/Male	20	3.69	5.42 (3.31,8.38)
20–39/Female	3	1.88	1.60 (0.33, 4.66)
40–59/Male	80	37.58	2.13 (1.69,2.65)
40–59/Female	11	6.92	1.59 (0.79, 2.85)
60–79/Male	29	19.40	1.49 (1.00,2.15)
60–79/Female	2	1.31	1.53 (0.18, 5.52)
Total	145	70.78	2.05 (1.73, 2.41)
Lung Cancer			
20–39/Male	1	0.06	16.55 (0.42, 92.21)
20–39/Female	0	0.03	0 (0, 118.43)
40–59/Male	14	4.16	3.37 (1.84, 5.65)
40–59/Female	4	0.63	6.37 (1.74, 16.31)
60–79/Male	11	2.90	3.80 (1.90, 6.79)
60–79/Female	0	0.24	0 (0, 15.55)
Total	30	8.02	3.74 (2.52, 5.34)
Anal Cancer			
20–39/Male	5	0.08	64.55 (20.98, 150.66)
20–39/Female	0	0.005	0 (0, 749.71)
40–59/Male	21	2.50	8.39 (5.19, 12.82)
40–59/Female	1	0.26	3.82 (0.10, 21.29)
60–79/Male	2	0.99	2.02 (0.24, 7.30)
60–79/Female	0	0.05	0 (0, 69.03)
Total	29	3.89	7.46 (4.99, 10.70)

^a^Expected number of NADMs are based on population incidence for that NADM

### Clinical characteristics associated with a NADM in individuals with HIV/AIDS

Amongst PLWHA receiving antiretroviral therapy, there were significant differences in the demographic and clinical characteristics of individuals diagnosed with an NADM when compared to individuals not diagnosed with NADM (Table 4). To further evaluate this effect size, univariate analysis identified six variables significantly associated with the development of a NADM amongst PLWHA receiving antiretroviral therapy: male gender, older age at start of antiretroviral therapy, lower nadir CD4 cell counts, presence of another NADM prior to the initiation of antiretroviral therapy, absence of HAART, and start of antiretroviral therapy in the early-HAART era (i.e. prior to 2000) (Table 5). In multivariate modeling, the use of HAART (i.e. triple antiretroviral therapy) was protective against development of a NADM compared to mono−/dual- antiretroviral drug therapy (aOR: 0.64, CI: 0.43 to 0.95). A higher nadir CD4 count was also found to be an independent protective factor for the development of a NADM (aOR: 0.61, CI: 0.41 to 0.93 for a CD4 of 200 cells/mm^3^ or greater). Being over the age of 50 (aOR: 4.03, CI: 3.05–6.06) and having history of NADM before initiating ART (aOR: 3.42, CI: 1.50–7.83) were both associated with the development of NADM among the sample of PLWHA in our study.

**Table 4:** Clinical characteristics of PLWHA on antiretroviral therapy by NADM status

Clinical characteristic	Individuals with NADM	Individuals without NADM^a^	*p*-value
	(*N* = 145)	(*N* = 4773)	
Gender			
Male	129 (88.97%)	3887 (81.44%)	0.021*
Female	16 (11.03%)	886 (18.56%)	
Age (years) at Start of Antiretroviral Therapy			
Under 30	8 (11.8%)	689 (14.44%)	<0.001*
30–39	41 (27.7%)	1891 (39.62%)	
40–49	41 (28.7%)	1478 (30.97%)	
50 or more	55 (31.8%)	715 (14.98%)	
History of Intravenous Drug Use			
Yes	42 (28.97%)	1724 (36.12%)	0.077
No	103 (71.03%)	3049 (63.88%)	
Nadir CD4 (cells/mm^3^)			
200 or more	28 (19.31%)	1372 (28.75%)	0.040*
100–199	48 (33.10%)	1382 (28.95%)	
50–99	28 (19.31%)	658 (13.79%)	
less than 50	41 (28.28%)	1361 (28.51%)	
DM Status			
Yes	7 (4.83%)	244 (5.11%)	0.878
No	138 (95.17%)	4529 (94.89%)	
Hepatitis C Status			
Positive	52 (35.86%)	1962 (41.11%)	0.085
Negative	75 (51.72%)	2035 (42.64%)	
Unknown	18 (12.41%)	776 (16.26%)	
NADM Prior to Start of Antiretroviral Therapy^b^			
Yes	6 (4.14%)	54 (1.13%)	0.008*
No	139 (95.86%)	4719 (98.87%)	
Use of HAART^c^			
Yes	112 (77.24%)	4163 (87.22%)	<0.001*
No	33 (22.76%)	610 (12.78%)	
Start of Antiretroviral Therapy^d^			
Early-HAART era (before 2000)	91 (62.76%)	2002 (41.94%)	<0.001*
Late-HAART era (2000 and after)	54 (37.24%)	2771 (58.06%)	

^a^Individuals with no NADM were represented by patients with either no cancer diagnosis (no ADM or NADM) or with an ADM only (but no NADM)

^b^Comparison groups were defined by individuals with diagnosis of NADM after initiation of antiretroviral therapy. There were 6 patients in the group “Individuals with NADM” that had a NADM prior to start of antiretroviral therapy and developed a second NADM during antiretroviral therapy. There were 54 patients in the group “Individuals without NADM” that had a NADM prior to antiretroviral treatment and did not develop an NADM during antiretroviral therapy

^c^All individuals were treated with antiretroviral therapy (monotherapy, dual therapy, or triple therapy); selected individuals were treated with HAART (triple therapy) according to guidelines at time of treatment

^d^The year cut-point was utilized to represent use of more efficient HAART starting in the year 2000

bolding and * indicates statistical significance at alpha 0.05

**Table 5:** Association of clinical characteristics with development of NADM in PLWHA receiving antiretroviral therapy^a^ (*n* = 4918)

Clinical characteristic	Unadjusted Odds ratio	Adjusted Odds ratio^b^
	(95% Confidence Intervals)	(95% Confidence Intervals)
Gender		
Male	1.00 (−-)	---
Female	0.59 (0.35–0.98)	
Age (years) at Start of Antiretroviral therapy		
Under 50	1.00 (−-)	1.00 (--)
50 or more	4.32 (3.08–6.06)	4.03 (3.05–6.06)
Nadir CD4 (cells/mm^3^)		
Less than 200	1.00 (−-)	1.00 (--)
200 or more	0.63 (0.42–0.95)	0.61 (0.41–0.93)
NADM Prior to Start of Antiretroviral Therapy^c^		
No	1.00 (−-)	1.00 (--)
Yes	4.87 (2.15–11.02)	3.42 (1.50–7.83)
Use of HAART^d^		
No	1.00 (−-)	1.00 (--)
Yes	0.76 (0.51–1.13)	0.64 (0.43–0.95)
Start of Antiretroviral Therapy^e^		
Early-HAART era (before 2000)	1.00 (−-)	---
Late-HAART era (2000 and after)	0.93 (0.31–1.34)	

^a^Amongst individuals with a NADM diagnosis compared to individuals without a NADM (represented by patients with either no cancer diagnosis [no ADM or NADM] or with an ADM only [but no NADM])

^b^Adjusted odds ratio includes all variables listed under ‘Clinical Characteristic’ column. C-statistic = 0.689; multi-collinearity was verified and all variance inflation factors were less than 2

^c^Only individuals with diagnosis of NADM after initiation of antiretroviral therapy were included in the comparison groups. There were 6 patients in the group “Individuals with NADM” that had a NADM prior to antiretroviral treatment and developed a second NADM during anti-retroviral therapy

^d^All individuals were treated with antiretroviral therapy (monotherapy, dual therapy, or triple therapy); selected individuals were treated with HAART (triple therapy) according to guidelines at time of treatment

^e^The year cut-point was utilized to represent use of more efficient HAART starting in the year 2000

### Survival of PLWHA diagnosed with a NADM

**Fig. 1:**
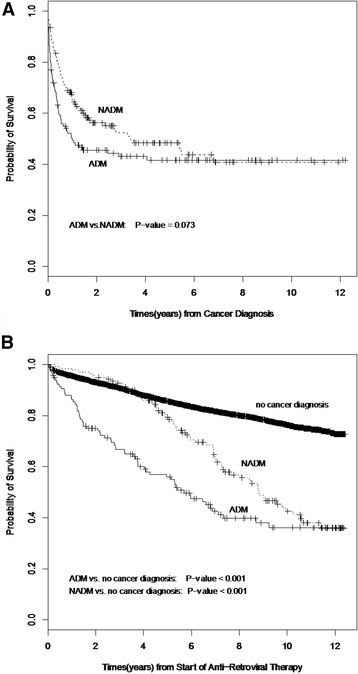
Overall survival of PLWHA receiving anti-retroviral therapy by cancer status from time of **a** cancer diagnosis and **b** start of antiretroviral therapy

Kaplan-Meier product limit estimates of cumulative overall survival were constructed for PLWHA in the study cohort (Fig. 1). Five patients were diagnosed with both a NADM and an ADM during the study period (i.e. during antiretroviral therapy) and were excluded from the categorical survival analysis. There was no significant difference in survival when comparing individuals diagnosed with an NADM compared to individuals diagnosed with an ADM prior to their cancer diagnosis (*p* = 0·073). Although a trend for improved early survival was observed in PLWHA diagnosed with an NADM compared to patients with an ADM, the survival probability of these two groups normalized at longer term follow-up. Amongst PLWHA receiving antiretroviral therapy, individuals with a NADM had a significantly lower survival compared to individuals without a cancer diagnosis (*p* < 0.001).

## Discussion

This is amongst the first study to report on NADM incidence in a Canadian PLWHA population. The study specifically focused on PLWHA that were receiving treatment during the current HAART era. Outcomes were based on linked, single-payer administrative data from the Bristish Columbia Cancer Registry (BCCR) and the Drug Treatment Program (DTP), which provides a comprehensive analysis of province-wide antiretroviral use and cancer incidence in British Columbia, Canada.

Within our study, 2.95% (145 of 4918) of patients developed a NADM, which represents a significantly higher risk when compared to individuals without HIV/AIDS in the general population (SIR 2.05, CI: 1.73 to 2.41). The types of NADMs most commonly diagnosed in our study population were generally consistent with other reports of HIV/AIDS populations [21–24]. The increased incidence of lung cancer that we observed in our study population has also been reported by other groups in PLWHA during both the pre-HAART and post-HAART eras [25–28]. Our study also found a significantly increased incidence of anal cancer in PLWHA when compared to the general population, particularly for males aged 20 to 39 years (SIR 64.55, C.I. 20.98 to 150.66) and aged 40 to 59 years (SIR 8.39, C.I. 5.19 to 12.82). This is a higher incidence than observations made by other groups in which the SIR for development of anal cancer ranged from 6.8 to 10.3 [29, 30]. An increase in lung and anal cancer incidence is known to be associated with shared risk factors, such as increased incidence of smoking in PLWHA [27, 31].

Along with the increased incidence in lung and anal cancers, we also observed that the majority of NADMs were represented by other cancer types, including cancers of the head and neck, liver, rectum, prostate, kidney and lymphatic system. These observations have important implications on screening and treatment protocols for PLWHA. Screening interventions for a variety of cancer types have been proven to be beneficial in the general population, and studies have been encouraging for adoption of specific cancer screening practices for PLWHA [28]. The increased incidence of other cancer types, not just lung and anal cancers, suggests that physician awareness and cancer screening are especially important for PLWHA.

To further address the observed increase in NADMs diagnosed in PLWHA, our study analyzed the association between NADM development, HAART utilization, and host clinical characteristics. Previous studies have hypothesized that HAART utilization is indirectly associated with decreased cancer incidence due to improvement of host immune surveillance and clearance of tumor cells [32]. HAART utilization is also associated with increased survival, which results in a more prolonged time interval for the development of NADMs. Furthermore, in recent years, cancer incidence may also be influenced by intensified screening practices and greater physician awareness of the HIV/AIDS population.

Our current study found that individuals undergoing treatment in the late-HAART era (2000 and later) did not have a significantly decreased incidence of NADM when compared to individuals that began therapy during the early-HAART era (prior to 2000). However, this may be due to the fact that many individuals would have changed treatment regimens during the later HAART era and as such, cannot be simply categorized by the date of initiation. However our work does suggest that although NADM incidence is increased when compared with the general population, the use of more effective and efficient HAART therapy is protective against the development of NADMs in PLWHA through immune reconstitution, and this is also supported by previous research [33–37]. This observation underscores the importance of early initiation of effective antiretroviral treatment, and implies that NADM incidence may be associated with immunological correlates in the HIV/AIDS population.

To further understand the mechanism by which HAART protects against NADM development, our present study found that a nadir CD4 level > 200 cells/mm^3^ was also protective against NADM development. Previous research has suggested that a higher percentage time with undetectable HIV RNA was protective against the development of NADMs, highlighting the importance of the host immune system in HIV clearance and NADM development [38]. Therefore, our observed association between CD4 levels and NADM development further supports the concept that HAART utilization may be protective due to its immune-restorative properties. These findings support early initiation of effective HAART therapy in order to maintain high CD4 levels. Furthermore, although the mechanisms that underlie the effects of HAART on the development of NADM in PLWHA are currently poorly understood, our observations suggest the importance of host immunological factors.

Previous studies have also suggested an association between HAART utilization and NADM incidence. In a study by Burgi et al. reporting on a cohort of 4144 HIV-positive individuals treated at military clinics in the United States between 1988 and 2003, the use of HAART was significantly associated with lower rates of NADMs [39]. Furthermore, studies from Australia and Europe have also reported higher CD4 levels and HAART utilization as being protective against NADM development [40, 41]. These reports are consistent with our observation and further suggest that the on-going immune-restorative effects of HAART may be beneficial in reducing the risk of NADM in PLWHA.

In our current study, we also observed that the development of a NADM not only resulted in a significantly reduced survival in PLWHA when compared to PLWHA without a cancer diagnosis, but also a post-cancer survival probability similar to individuals diagnosed with ADMs. The similarity in these survival outcomes suggests similarities in host reactions to all types of malignancies, which could be due to the specific immune factors required to clear the cancer cells. This would suggest that regardless of whether a cancer diagnosed in PLWHA is an ADM or NADM, protection against its development is impacted by adequate immune function, and therefore importantly mediated by HAART therapy.

The current study had several limitations, many of which are a consequence of its retrospective design. Evaluation of the number of NADMs and ADMs is highly dependent upon the accurate reporting of cancer in the provincial database. Furthermore, attempts to evaluate the impact of HAART on NADM diagnosis, represented by the absence or presence of HAART as well as the start date of antiretroviral treatment, are also potentially confounded by the changes that occurred in treatments over time. Moreover, there is considerable heterogeneity across NADM, and over time, and it can be difficult to infer direct clinical benefits of HAART uptake from epidemiological trends, although we suggest that overall we see a positive association between higher CD4 cell counts and cancer incidence. Conversely, there are notable differences between individuals receiving HAART therapy compared to individuals receiving mono−/dual- antiretroviral treatment, as we have controlled for duration of HIV infection but are unable to control for varying effects of therapeutic changes over time. Furthermore, age and CD4 level were included in the multivariate modeling as an objective measure of the health status of the study population.

## Conclusions

The current study provides a contemporary overview of cancer risk in a large population of PLWHA during the HAART era. Currently, NADMs are an important cause of morbidity in PLWHA. Utilization of HAART and its associated improved immune-restoration may be beneficial for these individuals. Unquestionably, the development of more effective cancer screening and prevention practices, that are not limited to anal and lung cancers, will be important in the future. Further study of cancer epidemiology in PLWHA is important and will lead to the development of strategies that improve outcomes for this unique population.

## Abbreviations

ADM: AIDS-defining malignancy
AIC: Akaike information criterion
ART: Antiretroviral Therapy
BC-CfE: British Columbia Centre for Excellence in HIV/AIDS
BCCR: British Columbia Cancer Registry
DTP: HIV/AIDS Drug Treatment Program
HAART: Highly active antiretroviral therapy
NADM: non-AIDS defining malignancy
PHN: Personal Health Number
PLWHA: People living with HIV/AIDS
SIR: Standardized incidence ratios
